# Peptide Modification Diminishes HLA Class II-restricted CD4^+^ T Cell Recognition of Prostate Cancer Cells

**DOI:** 10.3390/ijms232315234

**Published:** 2022-12-03

**Authors:** Bently P. Doonan, Shereen Amria, Jennifer R. Bethard, Narendra L. Banik, Jessica D. Hathaway-Schrader, Azizul Haque

**Affiliations:** 1 Department of Microbiology and Immunology, Medical University of South Carolina, 173 Ashley Avenue, Charleston, SC 29425, USA; 2 Hollings Cancer Center, Medical University of South Carolina, 96 Jonathan Lucas Street, Charleston, SC 29425, USA; 3 Childrens Research Institute, Medical University of South Carolina, 173 Ashley Avenue, Charleston, SC 29425, USA; 4 Department of Medicine, University of Florida, 1600 SW Archer Rd, Gainesville, FL 32608, USA; 5 Department of Neurosurgery, Medical University of South Carolina, 96 Jonathan Lucas Street, Charleston, SC 29425, USA; 6 Ralph H. Johnson Veteran Affairs Health Care System, 109 Bee St., Charleston, SC 29401, USA

**Keywords:** prostate cancer, prostate-specific membrane protein, HLA class II, cysteinylation, GILT, antigen presentation, CD4^+^ T cells

## Abstract

Prostate cancer poses an ongoing problem in the western world accounting for significant morbidity and mortality in the male population. Current therapy options are effective in treating most prostate cancer patients, but a significant number of patients progress beyond a manageable disease. For these patients, immunotherapy has emerged as a real option in the treatment of the late-stage metastatic disease. Unfortunately, even the most successful immunotherapy strategies have only led to a four-month increase in survival. One issue responsible for the shortcomings in cancer immunotherapy is the inability to stimulate helper CD4^+^ T cells via the HLA class II pathway to generate a potent antitumor response. Obstacles to proper HLA class II stimulation in prostate cancer vaccine design include the lack of detectable class II proteins in prostate tumors and the absence of defined class II specific prostate tumor antigens. Here, for the first time, we show that the insertion of a lysosomal thiol reductase (GILT) into prostate cancer cells directly enhances HLA class II antigen processing and results in increased CD4^+^ T cell activation by prostate cancer cells. We also show that GILT insertion does not alter the expression of prostate-specific membrane antigen (PSMA), an important target in prostate cancer vaccine strategies. Our study suggests that GILT expression enhances the presentation of the immunodominant PSMA_459_ epitope via the HLA class II pathway. Biochemical analysis showed that the PSMA_459_ peptide was cysteinylated under a normal physiologic concentration of cystine, and this cysteinylated form of PSMA_459_ inhibited T cell activation. Taken together, these results suggest that GILT has the potential to increase HLA class II Ag presentation and CD4^+^ T cell recognition of prostate cancer cells, and GILT-expressing prostate cancer cells could be used in designing cell therapy and/or vaccines against prostate cancer.

## 1. Introduction

Prostate cancer is the most commonly diagnosed male cancer in the United States [1,2]. The disease also accounts for considerable morbidity and mortality in this population, and it is the second leading cause of cancer-related deaths in men [1,2,3]. Treatment options for prostate cancer include radical prostatectomy, radiation (close-beam radiation and brachytherapy), hormone therapy, and chemotherapy [4,5,6,7]. Often, patients with even distant metastatic disease can lead a prolonged life by switching between therapeutic strategies, but for a subset of younger men with an aggressive disease, they exhaust these options early on and are left with clinical trials as their only viable option. Almost a decade ago, immunotherapy finally cracked through the treatment landscape and emerged as an attractive alternative approach to metastatic prostate cancer disease management with the FDA approval of Provenge (Sipuleucel-T, Dendreon) [8,9,10,11,12]. There was a lot of promise associated with this breakthrough, but unfortunately, the clinical utility and adoption of this therapy remained low, and it soon fell out of favor with improvements gained in second-generation androgen-blocking agents [13]. The lack of clinical adoption of Sipuleucel-T is multifactorial, but the few additional months of survival observed in clinical trials, discrepancies in trial design with the negative impact of apheresis in the control group, and many disparities in health care delivery are some of the reasons given [11,14,15]. The immunosuppressive tumor microenvironment (TME) and the inadequate response from helper T cells also make prostate cancer a difficult target for most immunotherapeutics. This has not been adequately analyzed in metastatic prostate tumors, lagging behind other tumor types. Recent strategies have employed immunotherapeutic approaches, such as; vaccines, and adoptive CAR-T cells, andimmune checkpoint inhibitors [16], but the clinical outcomes still need to be improved.

One issue responsible for these shortcomings in immunotherapy is the inability to stimulate both cytotoxic CD8^+^ and helper CD4^+^ T cells to generate a potent antitumor response [17,18,19]. Most current immunotherapy strategies focus on eliciting a cytotoxic immune response, ignoring the HLA class II pathway and CD4^+^ T cell activation. These treatments often fail to produce long-lasting antitumor immunity and the development of tumor-specific memory T cells [20,21,22]. Recently, our lab has shown that a natural triterpenoid, ganoderic acid DM (GA-DM), can activate antigen presentation in prostate cancer cells via the HLA class II pathway [19,23] and could be a candidate for future drug design for prostate cancer. We and others have also shown the importance of exploiting the HLA class II pathway in vaccine design in multiple cancer types [19,24,25,26,27]. Obstacles to proper class II stimulation in prostate cancer vaccine design include the lack of class II in prostate tumors and the lack of effective class II-specific prostate tumor antigens (Ags) [18,28,29]. Our laboratory has previously reported that, contrary to popular contention, prostate cancer cells and primary prostate tumors express detectable and functional HLA class II molecules capable of stimulating CD4^+^ T cells [30]. In the same study, we reported that through the alteration of cell culture conditions using hormone-enriched media, the expression of class II could be increased, similar to the results seen when prostate cancer cells are induced with interferon gamma (IFN-γ). However, IFN-γ stimulation broadly activates multiple pathways in the cancer cells and is by no means a specific HLA class II activator [31]. To subvert the unwanted side effects of IFN-γ treatment, the introduction of gamma–interferon inducible lysosomal thiol reductase (GILT), an enzyme found in professional antigen-presenting cells (APCs), could more specifically enhance HLA class II presentation in tumor cells [32,33].

Previous studies have shown that the induction of GILT in melanoma increases class II Ag processing, upregulates the expression of HLA class II molecules, and alters acidic proteases leading to enhanced CD4^+^ T cell stimulation [33,34]. We have also shown that GILT is necessary for the reduction of cysteinylated peptides, allowing for rapid presentation and loading onto HLA class II molecules [35]. Cysteinylated Ags form strong disulfide bonds that cannot be broken without the presence of a strong oxidoreductase [35,36]. These cysteinylated peptides are deficient in T cell stimulation, aiding in tumor-induced T cell tolerance. This issue is important, as many potential tumor Ags contain cysteine residues potentially susceptible to cysteinylation. Among them, the prostate-specific membrane antigen (PSMA) is important for prostate cancer [37]. PSMA is a type II membrane protein, and it has a unique three-part structure: a 19-amino acid internal portion, a 24-amino acid transmembrane portion, and a 707-amino acid external portion [38]. PSMA has known enzymatic activities and acts as a glutamate-preferring carboxypeptidase. PSMA is expressed by virtually all prostate cancers, and its expression is further increased in poorly differentiated, metastatic, and hormone-refractory carcinomas, making it a very attractive target [39,40]. In PSMA, the HLA class II-restricted immunodominant peptide (PSMA)_459_ (NYTLRVDCTPLMYSL) contains a central cysteine residue which may alter its ability to stimulate T cells [41]. Whether GILT’s insertion in prostate cancer cells alters Ag’s processing and presentation to prostate cancer cells has yet to be determined. In this study, we show for the first time that GILT insertion into prostate cancer cells directly enhances the HLA class II-restricted presentation of cysteinylated peptides and results in increased CD4+ T cell activation by prostate cancer cells.

## 2. Results

### 2.1. IFN-γ Treatment Induces GILT in Human Prostate Cancer Cells

**Figure 1:**
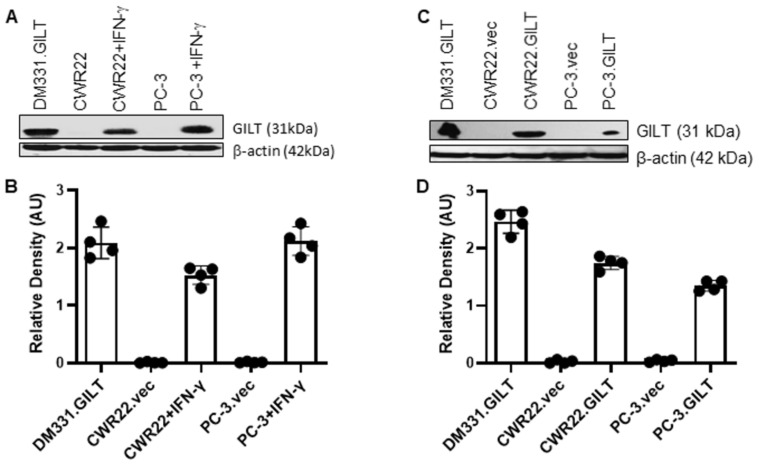
Induction of GILT in human prostate cancer cells by treatment with IFN-γ and insertion of GILT in prostate cancer cells with GILT cDNA. (**A**) Human prostate cancer cell lines CWR22Rv1 and PC-3 were treated with IFN-γ for three days, followed by washing and Western blot analysis for GILT protein expression. β-actin was used as a loading control. (**B**) Quantitative analysis of protein bands from (**A**) by ImageJ software. (**C**) CWR22Rv1 and PC-3 cells were transfected with GILT cDNA and analyzed by Western blotting for GILT protein expression. A melanoma cell line previously transfected with GILT cDNA (DM-331.GILT) was used as a control. β-actin was used as a loading control. (**D**) Quantitative analysis of protein bands from (**C**) by ImageJ software. Data showed that IFN-γ-treated and GILT-transfected CWR22Rv1 and PC-3 cells contained detectable levels of GILT proteins.

Many cancers, including prostate cancer, downregulate the HLA class II expression and also display a reduced Ag processing capability and lack GILT expression, a key enzyme involved in HLA class II peptide generation. The induction of GILT in prostate cancer cells may have significant effects on Ag processing and presentation via the HLA class II pathway. To examine whether prostate cancer cells express GILT, CWR22Rv1 and PC-3 cell lines were treated with or without IFN-γ (800 U/mL) for 72 h (Figure 1A). Western blot analysis showed that IFN-γ treatment induced detectable levels of GILT in CWR22 and PC-3 cells. As stated previously, IFN-γ treatment also induces the expression of many unwanted proteins, so in order to more directly examine the role of GILT, it was necessary to insert GILT into prostate cancer cells. A lipofectamine transfection reagent was used to insert GILT CDNA in CWR22 and PC-3 prostate cancer cells (Figure 1B). Western blot analysis was used to confirm the GILT expression in CWR22Rv1 and PC-3 prostate cancer cell lines versus vector transfected cell lines, which express a puromycin resistance gene but no GILT cDNA. The melanoma cell line DM-331.GILT was used as a control. These results support the notion that prostate cancer cells can be induced to express GILT, which may influence the activation of CD4+ T cells via the HLA class II pathway.

### 2.2. GILT Expression Did Not Alter PSMA Levels in Prostate Cancer Cells

**Figure 2:**
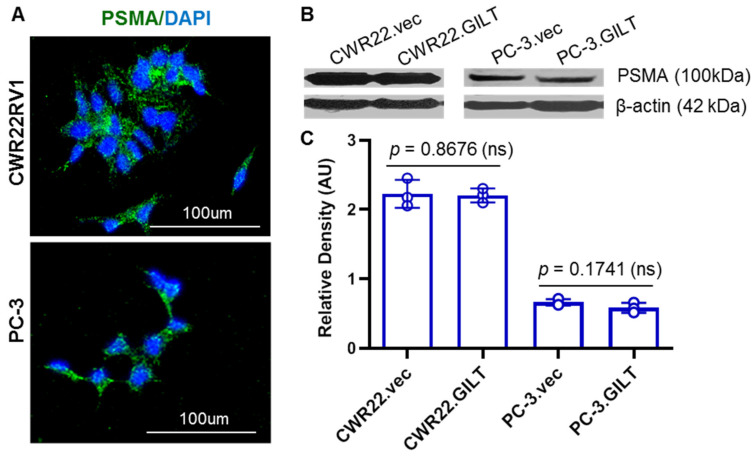
GILT insertion did not influence PSMA protein expression in human prostate cancer cells. (**A**) The expression of PSMA was tested in CWR22Rv1 and PC-3 prostate cancer cell lines by immunofluorescent staining (green) with the anti-PSMA antibody, followed by confocal microscopy. (**B**) CWR22Rv1 and PC-3 cells transfected with either empty vector or GILT cDNA and analyzed by Western blotting for PSMA protein expression. β-actin was used as a loading control. (**C**) Densitometric analysis of protein bands obtained from CWR22Rv1.vec, CWR22Rv1.GILT, PC-3.vec, and PC-3.GILT showed no significant changes in PSMA protein expression following GILT transfection. Data are representative of three separate experiments.

We examined the PSMA expression in two well-studied prostate cancer cell lines by confocal microscopy (Figure 2A). Both CWR22 and PC-3 cells expressed PSMA (green fluorescence), where DAPI was used for nuclear staining. The expression of PSMA in CWR22 and PC-3 cells was also confirmed by Western blot analysis, where β-actin was used as a loading control (Figure 2B). To study whether GILT expression influences the PSMA protein expression in prostate cancer cells, we next examined CWR22 and PC-3 cells with or without GILT. Western blot analysis showed that GILT insertion did not significantly alter PSMA protein levels in CWR22 and PC-3 cells (Figure 2B,C). The androgen-dependent prostate cancer cell line CWR22Rv1 constitutively expresses high levels of PSMA as shown through Western blotting and quantitative analyses (Figure 2C). These data suggest that GILT insertion does not alter the PSMA protein expression, although GILT’s function in PSMA processing and epitope generation may influence immune responses in prostate cancer cells.

### 2.3. Prostate Cancer Cell EHLA Class II Protein and GILT Insertion Did Not Markedly Alter HLA Protein Expression on the Cell Surface of CWR22 and PC-3 Cells

**Figure 3:**
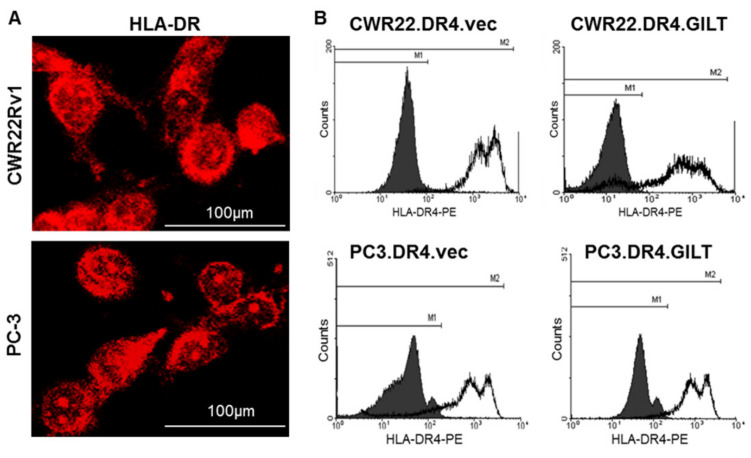
GILT insertion did not significantly alter HLA class II protein expression in human prostate cancer cells. (**A**) The expression of HLA-DR was tested in CWR22Rv1 and PC-3 prostate cancer cell lines by immunofluorescent staining (red) with the anti-HLA-DR antibodies (L243), followed by confocal microscopy. (**B**) CWR22Rv1, CWR22Rv1.GILT, PC-3, and PC-3.GILT cells were transduced with HLA-DR4 by retroviral transduction and stained with anti-DR4 antibodies (359-F10). Cells were then analyzed by flow cytometry for HLA-DR4 expression. IN-1 was used as an isotype control. Data are representative of three separate experiments.

CWR22 and PC-3 cells were cultured in ΔCCS, containing media as described in the Materials and Methods, and they were stained (red) with antibodies against HLA-DR (L243). Confocal microscopic analysis detected the HLA-DR protein expression in both CWR22Rv1 and PC-3 cells (Figure 3A). Given GILT’s ability to enhance the HLA-DR protein expression in melanoma cells, we next examined whether GILT insertion had any effects on the HLA class II protein expression. CWR22Rv1 and PC-3 cells with or without GILT were then transduced with the HLA-DR4β and analyzed by flow cytometry for HLA-DR4 molecules on the cell surface (Figure 3B). These studies showed that CWR22Rv1 and PC-3 cells expressed detectable HLA-DR4 proteins, and GILT insertion did not significantly alter HLA-DR4 expression on the cell surface.

### 2.4. GILT Expression Enhances the HLA Class II Mediated Antigen Presentation in PSMA-Expressing Prostate Cancer Cells

**Figure 4:**
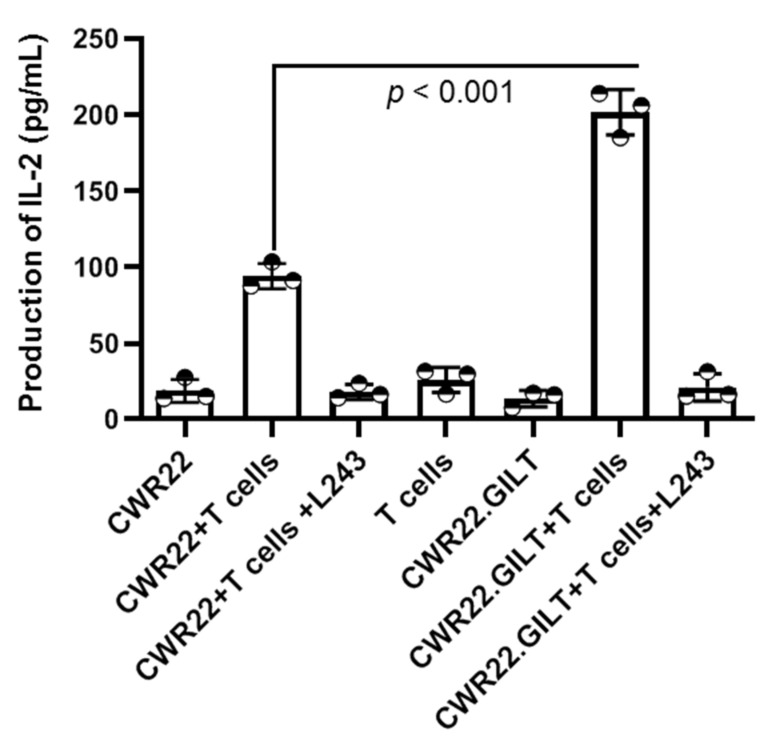
GILT expression enhances CD4^+^ T cell recognition of prostate cancer cells. HLA-DR-expressing prostate cancer cell lines CWR22Rv1.vec and CWR22Rv1.GILT were cocultured with PSMA-specific CD4^+^ T cells raised from an HLA-DR-positive healthy donor in the presence or absence of anti-class II antibodies (L243) for 48 h. Human IL-2 ELISA was performed to quantitate the amount of IL-2 present in the culture supernatant and was expressed as pg/mL. Data are representative of at least three separate experiments.

To examine the effect of GILT on the Ag presenting capacity of the prostate cancer cell line CWR22Rv1 to CD4^+^ T cells, CWR22 and CWR22.GILT cells were cocultured with HLA-DR-restricted PSMA-specific CD4^+^ T cells for 48 h. Analysis of IL-2 in the cell supernatant suggests that CWR22 cells can functionally present PSMA to CD4^+^ T cells (Figure 4). The addition of the HLA class II antibody (L243) in the assay blocked PSMA presentation by CWR22 cells, suggesting the presentation of PSMA Ag to T cells was HLA-DR-restricted. A closer look at the data showed that the GILT expression in CWR22 cells significantly increased T cell activation as compared to CWR22 cells lacking GILT. These data suggest that GILT insertion in prostate cancer cells enhances the HLA class II-restricted Ag presentation to CD4^+^ T cells. The data also suggest that the insertion of GILT in prostate cancer cells could make them a better target for CD4^+^ T cells.

### 2.5. Differential Presentation of PSMA Peptides to CD4^+^ T Cells: Role of GILT in Enhanced Presentation

**Figure 5:**
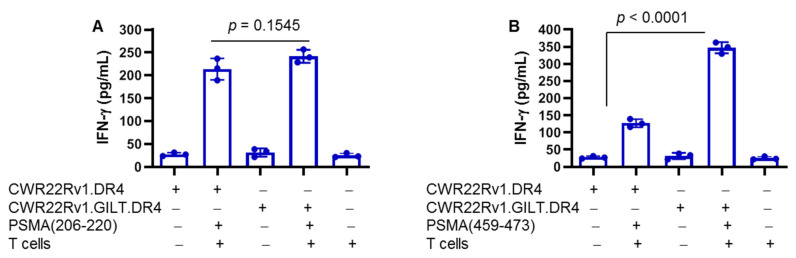
CD4^+^ T cell recognition of PSMA peptides on prostate cancer cells. CWR22Rv1 and CWR22Rv1.GILT cells were retrovirally transduced with HLA-DR4 as described in the methods. CWR22Rv1.DR4 and CWR22Rv1.GILT.DR4 cells were then incubated with the PSMA_(206–220)_ (**A**) and PSMA_(459–473)_ (**B**) peptides (20 μm) overnight at 37 °C and were washed and fixed with 1% paraformaldehyde. Cells were washed again to remove paraformaldehyde and were cocultured with the PSMA_206–220_ and PSMA_459–473_ peptide-specific CD4^+^ T cells for 48 h. The production of IFN-γ was quantitated using ELISA and expressed as pg/mL. Data are representative of three separate experiments.

To study PSMA peptide presentation by CWR22 cells, two peptides, PSMA_206–220_ and PSMA_459–473_, were used in the T cell assay. PSMA peptide-specific CD4^+^ T cells were generated by repeated stimulation of PBMCs from healthy HLA-DR4 individuals. These T cells were analyzed by flow cytometry for CD4 expression (data not shown). CWR22.vec.DR4 and CWR22.GILT.DR4 cells were incubated with PSMA_206–220_ and PSMA_459–473_ peptides (20 μm) overnight and were washed and fixed with 1% paraformaldehyde as previously described [42,43]. Cells were then cocultured with PSMA_206–220_ and PSMA_459–473_ peptide-specific CD4+ T cells for 48 h. The production IFN-γ in the cell supernatant was analyzed by ELISA. Results showed that both CWR22.vec and CWR22.GILT cells efficiently presented the PSMA_206–220_ peptide to CD4^+^ cells (Figure 5A). In contrast, CWR22.DR4 cells lacking GILT were unable to efficiently present the PSMA_459–473_ peptide to CD4^+^ T cells (Figure 5B). GILT insertion in CWR22.DR4 cells (CWR22.DR4.GILT) significantly increased the stimulation of CD4^+^ cells as compared to CWR22.DR4 cells lacking GILT. These data suggest that GILT insertion in CWR22.DR4 cells enhances the PSMA_459–473_ peptide presentation to CD4^+^ T cells and stress the important role that GILT plays in the HLA class II pathway.

### 2.6. Immunodominant PSMA_459_ Peptide Contains a Cysteine Residue Which May Become Oxidized and Disrupt Peptide Presentation by CWR22 Cells

**Figure 6:**
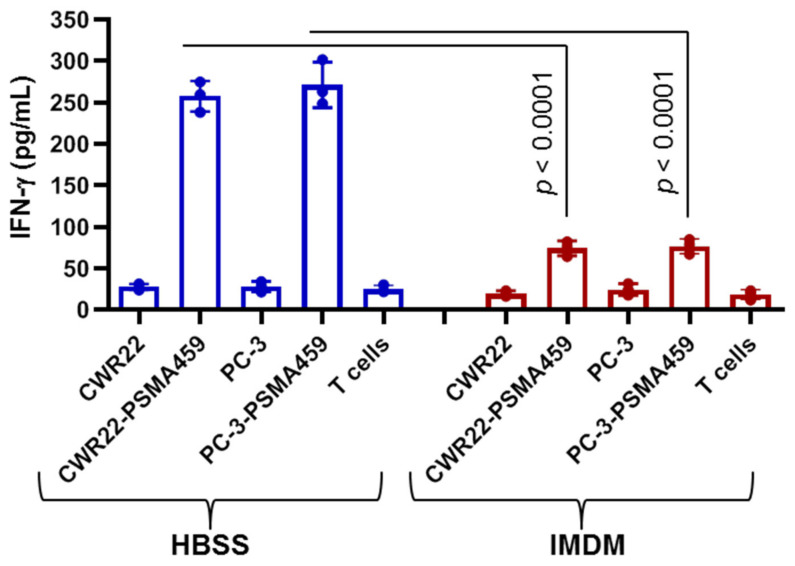
Cysteinylation of the PSMA459 peptide reduces CD4^+^ T cell recognition of prostate cancer cells. HLA-DR4-expressing CWR22Rv1.DR4 and PC-3.DR4 cells were incubated with the PSMA_459–473_ peptide (20 µm) in either HBSS buffer or IMDM media overnight at 37 °C and were washed and fixed with 1% paraformaldehyde. Cells were washed again and were cocultured with the PSMA_459–473_ peptide-specific CD4^+^ T cells for 48 h. The production of IFN-γ was quantitated using ELISA and expressed as pg/mL as described. Data are representative of three separate experiments.

As stated previously, GILT’s main role is in the reduction of oxidized or cysteinylated peptides in the endosomal and lysosomal compartments prior to loading onto HLA class II proteins. There are many prostate cancer Ags susceptible to cysteinylation reactions, most notably the immunodominant PSMA_459_ peptide which contains cysteine. Therefore, we examined the PSMA_459_ peptide presentation in media/buffer with or without cysteine. In the first batch, we incubated CWR22.DR4 cells with the PSMA_459_ peptide (20 μm) in HBSS, which did not contain cystine, overnight at 37 °C. In the second batch, we incubated CWR22.DR4 cells with the PSMA_459_ peptide (20 μm) in complete T cell media, which contained physiological concentrations of cystine (≅290 μM), overnight at 37 °C. Cells were washed, fixed, and cocultured with the PSMA_459_ peptide-specific CD4^+^ T cells for 48 h (Figure 6). Analysis of IFN-γ in the cell supernatant showed that CWR22 cells efficiently presented that PSMA_459_ peptide to CD4^+^ T cells when tested in HBSS lacking cysteine. In contrast, CWR22 cells minimally presented the PSMA_459_ peptide to CD4^+^ T cells when tested in media containing cysteine. These results suggest that the PSMA_459_ peptide may become cysteinylated in the presence of oxidized cysteine, and the cells are incapable of optimum presentation and T cell stimulation potentially limiting its future use in immunotherapy vaccine design.

### 2.7. Immunodominant PSMA_459_ Peptide Becomes Cysteinylated under a Normal Physiological Concentration of Cysteine in Media

**Figure 7:**
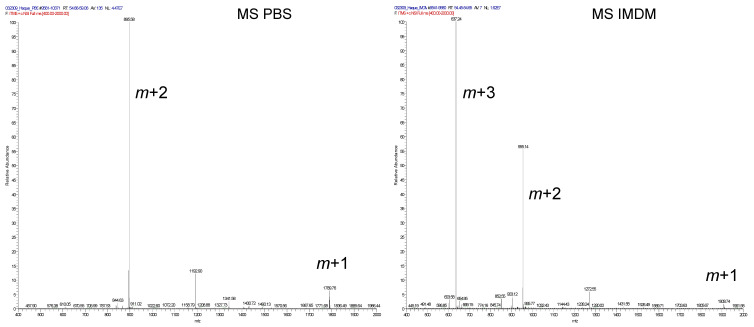

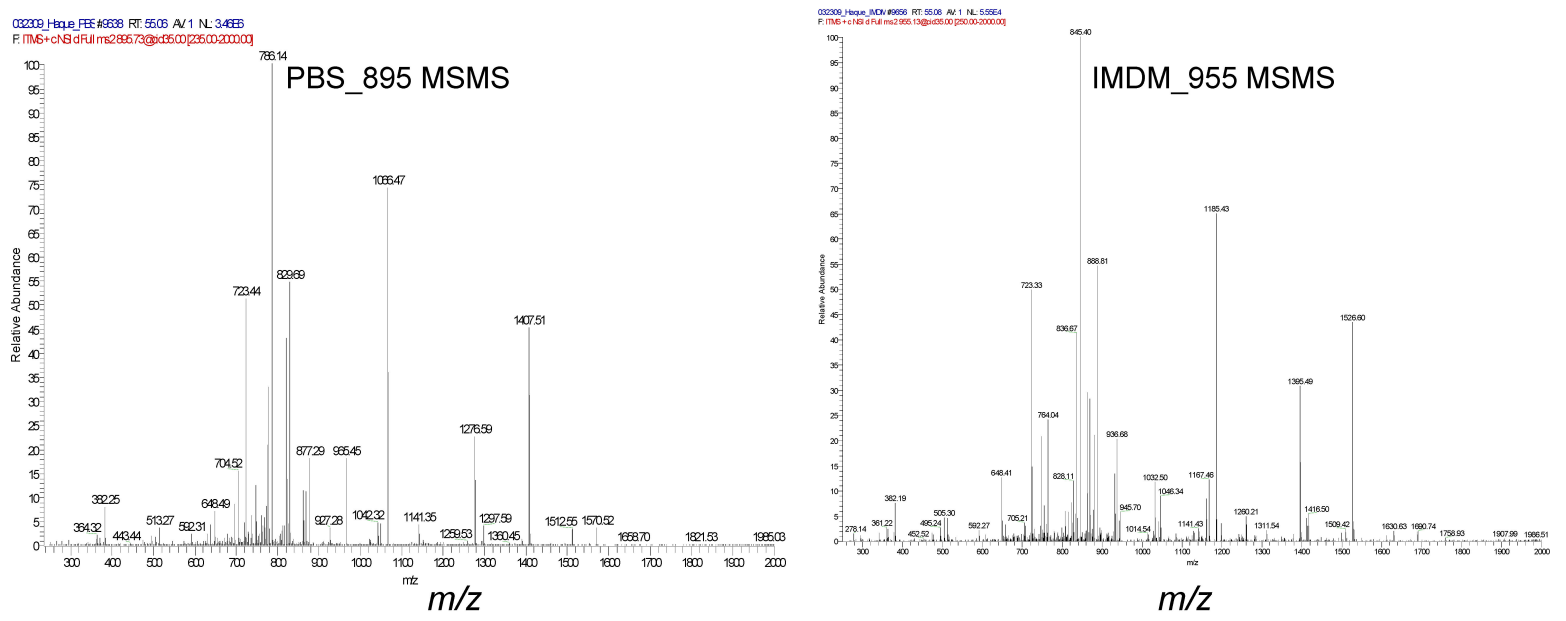
Mass spectroscopic analysis of the PSMA459 (NYTLRVDCTPLMYSL) peptide. The PSMA_(459–473)_ peptide was dissolved in PBS and culture medium (IMDM) which contains biological levels of cystine (0.29 mM). The PSMA459 peptide fragmentation in PBS was consistent with the calculated molecular mass of 1789.7 (upper panel). Fragmentation of the PSMA_459_ peptide after incubation in IMDM media indicated a mass of 1908.7, consistent with cysteinylation. MSMS analyses in PBS and IMDM are shown in the lower panels. These data suggest that the PSMA_459_ peptide becomes cysteinylated in media containing a physiological concentration of cystine.

Mass spectral analysis of the PSMA_459_ peptide dissolved in PBS revealed a single monomeric species (molecular mass, 1789.7) with a residue of Cys^466^ existing as a free sulfhydryl (Figure 7, top-left panel). However, upon incubation of the peptide in tissue culture medium (IMDM), the peptide was modified by a cysteine adduct yielding the 1908.7 molecular mass cysteinylated species (Figure 7, top-right panel). Mass spectral analysis failed to reveal any significant amounts of dimeric peptide as may be predicted based upon the disulfide formation. The peptide sequence and the presence of the modified cysteine were confirmed by collision-induced dissociation (Figure 7, bottom panels). These results confirm that the immunodominant PSMA_459_ peptide becomes cysteinylated under a normal physiological concentration of cysteine in media, potentially inhibiting T cell activation.

## 3. Discussion

Prostate cancer poses an ongoing problem in the western world accounting for significant morbidity and mortality in the male population [2,44,45]. Early detection and current therapeutic interventions are effective in treating the majority of prostate cancer cases; however, a portion of prostate cancer patients progress beyond the help of conventional therapy [6,45]. For these people, immunotherapy in the form of checkpoint inhibition in high-tumor mutational burden patients and the under-utilized DC therapy Sipuleucel-T are options [8,9,14,46]. Despite the initial success of this therapy, further DC vaccines have failed to achieve the expected outcome, and issues with trial design, the overall ability to induce an immune response but not a significant antitumor response, and cost have limited the clinical application of Sipuleucel-T. In addition, successful immunotherapy intervention has yet to deliver long-term disease relief, providing a limited survival time before the disease relapses and is ultimately fatal [47]. The shortcomings of current immunotherapy strategies are due in part to their focus on inducing a robust CD8^+^ cytotoxic T cell response, while ignoring the supportive CD4^+^ helper T cell response [48,49,50]. Recent evidence suggests that CD4^+^ T cells play a vital role in CD8^+^ T cell support and in the direct killing of tumors through the release of cytotoxic cytokines [20,51,52]. Thus, the stimulation of CD4^+^ T cells via the HLA class II pathway is of direct importance in antitumor immunity and should be addressed in future vaccine designs.

CD4^+^ T cells interact with bound peptides presented on HLA class II molecules by professional APCs and, to a lesser extent, directly by tumors [21,52]. Our recent study challenged the previously held notion that prostate cancer cells express no surface HLA class II, showing that both primary prostate tumor and prostate cancer cell lines express stable surface HLA class II capable of stimulating T cells. We also showed that the manipulation of the growth conditions of prostate cancer cells increases the expression of surface HLA class II to a similar degree as IFN-γ treatment. However, IFN-γ treatment also stimulates unwanted effects within the cells, so an alternative method of HLA class II stimulation is needed. Previous studies in professional APCs showed a central role for GILT in processing Ag peptides for proper HLA class II presentation [35]. Not surprisingly, GILT is almost completely absent from cancer cells, including prostate cancer, contributing to the poor processing and presentation of endogenous Ags for CD4^+^ T cell stimulation.

Our current investigation shows, for the first time, that GILT’s insertion into prostate cancer cells enhances Ag presentation via the HLA class II pathway. An increased HLA-DR expression does not always correlate to increased T cell activation, so experiments were performed to determine GILT’s ability to increase endogenous Ag presentation. Using the CWR22Rv1 cell line, which endogenously expresses high levels of the prostate tumor Ag PSMA, we examined any effect GILT insertion had on T cell activation. When cocultured with healthy donor T cells, GILT-positive CWR22Rv1 cells significantly increased T cell activation versus their vector counterparts based on IL-2 production. Similarly, experiments using GILT-transfected PC-3 prostate cancer cells mirrored the observations seen in the CWR22Rv1 cell lines (data not shown). Through co-incubation with an HLA-DR-blocking antibody, L243, this response was muted, returning T cell activation to control levels. This suggests that GILT insertion increased the HLA-DR-specific T cell activation in prostate cancer cells.

In addition to GILT’s role in Ag presentation, previous studies have shown GILT’s central role in Ag processing through boosting acid cathepsin activity and breaking disulfide bonds formed during cysteinylation [34]. Cysteinylation is an important aspect of peptide modification that is often overlooked. Our own study suggests that cysteinylated peptides require additional endosomal processing prior to loading onto HLA-DR molecules in order for effective T cell stimulation to take place [53]. Given the lack of GILT expression in prostate cancer, cysteinylated peptides would not be reduced and presented to CD4^+^ T cells displaying an altered, nonfunctional peptide that could induce T cell tolerance. One important prostate cancer Ag that is potentially susceptible to cysteinylation is PSMA. PSMA is a non-soluble type 2 integral membrane protein with carboxypeptidase activity, expressed on the apical surface of endothelial cells [40,54]. PSMA is weakly expressed in normal prostate tissue but overexpressed in metastatic prostate cancer and is present in >80% of men with prostate cancer [37,40,55]. PSMA expression is also increased in androgen deprivation and is highest in high-grade and castration-resistant prostate cancer (CRPC) [55,56], and it could be used as a target for metastatic prostate cancer. Recent studies have focused on PSMA as it has been the target of multiple immunotherapy strategies including protein/peptide vaccines and as a target for monoclonal antibodies [55,57,58,59,60]. As stated previously, the CWR22Rv1 cell line expressed high levels of endogenous PSMA, easily detectable through confocal microscopy and Western blotting. We next examined any effect GILT has on PSMA expression. Results showed that GILT expression did not alter endogenous PSMA expression in the CWR22Rv1 cell lines, nor did it influence mutant PSMA expression in PC-3 cells, which expressed low levels of mutant PSMA. These finding support the inclusion of GILT in vaccine strategies targeting PSMA in prostate cancer cells. However, the question of PSMA becoming cysteinylated and further limiting Ag presentation remains unknown.

Much work has been conducted to determine the specific immunoprevalent and immunodominant epitopes of PSMA [41,61]. From this work, the HLA-DR-restric-restricted immunodominant PSMA_459_ peptide has been identified [41]. Similar to many other potential PSMA peptides, PSMA_459_ contains a cysteine residue that is potentially susceptible to cysteinylation. To determine if the PSMA_459_ peptide becomes cysteinylated, we incubated synthetic recombinant peptide in culture medium with biological concentrations of the amino acid cystine, which preferentially binds to cysteine forming a dimer. Following three hours of incubation in this medium or in cystine-free PBS, mass spectral analysis was used to determine any alteration in the peptide. Results confirm PSMA_459_ becomes cysteinylated in the presence of cystine, which may inhibit T cell activation. To test this hypothesis, PSMA_459_ peptide was incubated in cystine-containing media, then cocultured with HLA-DR4-specific healthy donor T cells. Results confirm that the cysteinylated PSMA_459_ peptide significantly inhibits T cell proliferation. It is possible that the additional T cell activation observed in the PSMA-positive CWR22Rv1.GILT cell line could be due to a reduction in cysteinylation in the endosomal compartment by GILT. However, in the absence of GILT in protein/peptide vaccine strategies using the PSMA_459_ peptide, cysteinylation reactions could be avoided by altering the peptide through substitution of the cysteine residue. Overall, our study suggests that a modified peptide ligand for the PSMA_459_ peptide may be better equipped in stimulating T cells in protein/peptide-based vaccine strategies than the naturally occurring peptide.

In recent years, immunotherapy has emerged as a real option in treating late-stage metastatic prostate cancer patients. However, issues with immunotherapy design must be resolved if long-term clinical outcomes are to be expected. These include the incorporation of strategies which target both CD4^+^ helper T cells along with CD8^+^ cytotoxic lymphocytes. Proper CD4^+^ stimulation requires efficient HLA class II Ag presentation by both professional and nonprofessional APCs, including prostate cancers themselves. Here we show for the first time the ability of GILT to enhance the HLA-DR-mediated T cell activation in prostate cancer cells. These effects could be exploited in whole-cell cancer vaccine designs utilizing GILT transfection ex vivo prior to re-administration, similar to the method employed in the GVAX vaccines. HLA class II Ag presentation also requires effective Ag processing to effectively activate T cells; this includes the processing of cysteinylated peptides. We have also shown here that the immunodominant PSMA_459_ peptide becomes cysteinylated under normal physiological conditions leading to T cell inhibition. This inhibition could be overcome through the substitution of the cysteine residue with similar amino acids, creating altered peptide ligands that do not require further endosomal processing prior to loading onto class II complexes. These ligands could then better stimulate T cells when used in protein/peptide or loaded DC-based vaccine strategies. Together, these results represent potential new directions in improved prostate cancer immunotherapeutics, which should be explored in future vaccine designs.

## 4. Methods and Materials

### 4.1. Cell Lines and Culture Conditions

Prostate cancer cell lines CWR22Rv1 and PC-3 were grown in RPMI medium containing 10% fetal bovine serum (FBS) (HyClone, Logan, UT, USA), 50 U/mL of penicillin, 50 µg/mL of streptomycin (Mediatech Inc, Herndon, VA, USA), and 260 µg/mL of L-glutamine (Mediatech) [30]. The PC-3 cell line used in this study expresses a mutant form of PSMA. Prostate cancer cell lines were cultured in 100 × 20 mm sterile culture dishes (Corning Incorporated, Lowell, MA, USA) in a humidified incubator with 5% CO_2_ at 37 °C. Various concentrations of IFN-γ (Calbiochem, San Diego, CA, USA) were used to treat prostate cancer cells prior to specific experiments outlined later. Prostate cancer cell lines PC-3 and CWR22Rv1 were transfected using lipofectamine (Invitrogen, Waltham, MA, USA) transfection reagent with a GILT cDNA plasmid containing a puromycin resistance gene or with a vector cDNA plasmid containing the same drug resistance gene. The GILT expression was determined through Western blot analysis, using the B-cell line Frev which constitutively expresses high levels of GILT as a positive control. The B-cell line Frev was grown in Isocove’s Modified Dulbecco’s Medium (IMDM) (Mediatech) containing 10% FBS (HyClone), 50 U/mL of penicillin, 50 µg/mL of streptomycin, and 260 µg/mL of L-glutamine. Healthy primary peripheral blood mononuclear cells (PBMCs) were extracted from donor patient blood using a Ficoll–Paque Plus (Amersham Bioscience, Piscataway, NJ, USA) gradient following the manufacturer’s protocol. BPD PBMCs and the HLA-DR*0401 DLN T cells were cultured in complete RPMI medium containing 10% FBS, 50 U/mL of penicillin, 50 µg/mL of streptomycin, 260 µg/mL of L-glutamine, and 30.8 µM of β-mercapthoethanol (Invitrogen, Carlsbad, CA, USA).

### 4.2. Cell Transduction and Transfection

The prostate cancer cell lines CWR22 and PC-3 were transduced using retroviral vectors for the constitutive expression of HLA-DR4 (DRB1*0401) with linked drug selection markers for hygromycin and histidinol resistance [53]. The expression of surface HLA-DR4 complexes on cells was confirmed by flow cytometric analysis using the DR4-specific mAb, 359-F10 [62]. Prostate cancer lines CWR22 and PC-3 and the DM-331 melanoma cell line were transfected with an empty vector or GILT cDNA to generate CWR22.GILT, PC-3.GILT, and DM-331.GILT lines [53,63]. The expression of GILT was confirmed by Western blot analysis.

### 4.3. Peptides

The human PSMA peptides, PSMA_206–220_ (sequence: GKVFRGNKVKNAQLA) and PSMA_459_ (sequence: NYTLRVDCTPLMYSL), were produced using Fmoch technology and an Applied Biosystems Synthesizer [30,35]. These peptides were analyzed using reverse-phase high-performance liquid chromatography purification and mass spectroscopy, indicating a purity > 99%.

### 4.4. Antibodies

Human primary antibodies were used in this study to detect the protein expression of HLA-DRβ (XD5), HLA-DRαβ (L243), PSMA, GILT, and actin [36,42,64]. Antibodies used were acquired from the Dr. Janice Blum’s laboratory (Indiana University) or from Santa Cruz Biotechnologies (CA, USA). The secondary antibodies used, goat anti-mouse, anti-rabbit, or anti-goat IgG, were conjugated with horseradish peroxidase (Pierce, Rockford, IL, USA; Santa Cruz Biotech, CA, USA).

### 4.5. Flow Cytometry

Prostate cancer cell lines CWR22.DR4, CWR.22.DR4.GILT, PC-3, and PC-3.DR4.GILT were grown in RPMI plus ΔCCS and stained with an HLA-DR4-specific antibody (359-F10), followed by a secondary antibody labeled with fluorescein isothiocyanate (FITC). Samples were analyzed on a FACScan using the CellQuest software (BD Bioscience, Mountain View, CA, USA) [30]. Background fluorescence was evaluated using an irrelevant isotype-matched antibody IN-1 as described [30].

### 4.6. Western Blot Analysis

The prostate cancer cell lines (CWR22.vec, CWR22.GILT, PC-3.vec, and PC-3.GILT) and the melanoma cell line (DM-331.GILT) were lysed using lysis buffer (10 mM Trizma base, 150 mM NaCl, 1% Triton-X 100, 10 µM PSMF, and 10 µM TLCK). Equal protein concentrations between the vector and GILT cell lines were separated on 4–12% Bis/Tris NuPage gels in 2-(N-morpholino) ethenesulfonic acid (MES) buffer (Invitrogen) [33]. Blots were transferred to nitrocellulose membranes (Fischer Scientific, Hampton, NH, USA) and analyzed for PSMA and GILT. Blots were incubated with chemiluminescent SuperSignal (Pierce, Appleton, WI, USA) and exposed to an autoradiography film (Blue Devil, Barcelona, Spain) [30].

### 4.7. Confocal Microscopy

CWR22 and PC-3 cells were cultured on glass coverslips (Cat#12-545-80, Fisher Scientific Co) and incubated at 37 °C for 24 h. Cells were fixed with a 50/50 acetone/methanol mixture for 10 min at room temperature. Cells were then permeabilized with 0.1% Triton-X100 for 15 min, followed by blocking with 5% normal serum for 10 min, then incubated with PSMA (Santa cruz, Dallas, TX, USA) or HLA-DR (L243) antibodies at 37 °C for 1 h. Following incubation, cells were washed twice with 1% BSA (cat# 2930, OmniPur, EMD, Burlington, MA, USA) in PBS and incubated with Invitrogen Alexa Fluor 488 (green for PSMA) conjugated donkey anti-goat Ig (Santa Cruz Biotechnology) and Alexa Fluor 594 (red for HLA-DR) conjugated donkey anti-mouse Ig (Santa Cruz) for 1 h. The slides were mounted in fluorescent mounting medium G (South Biotechnology, Inc, Birmingham, AL, USA), observed with a 63× N.A.1.4 oil immersion objective lens, and analyzed by a Leica TCS SP5 confocal laser scanning microscope using the Las-AF software (Leica Lasertechnik, Wetzlar, Germany) [34].

### 4.8. Sample Preparation for LC MS/MS

Cell elusions were reduced with DTT and alkylated with 55 mM iodoacetamide. The protein was digested with 100 ng of trypsin (Sigma, proteomics grade) overnight at 37 °C. The digested sample was then desalted using a C18 ZipTip (Millipore, St. Louis, MO, USA) following the manufacturer’s protocol. The eluent was dried down, and peptides were resuspended in 5 µL of mobile phase A (98% water, 2% acetonitrile, 0.1% formic acid) and transferred to an auto sampler vial for LC-MS/MS analysis as described [35].

### 4.9. Mass Spectrometry

The peptide PSMA459 was incubated in DPBS or IMDM media for 3 h, and samples were analyzed via liquid chromatography (LC)-electrospray ionization (ESI)-tandem mass spectrometry (MS/MS) on a linear ion trap mass spectrometer (LTQ, Thermo Finnigan, San Jose, CA, USA) coupled to an LC Packings nano LC system. A 75-micron C-18 reversed-phase LC column (Microtech Scientific, Orange, CA, USA) was utilized with a 60 min gradient from 2% acetonitrile and 0.2% formic acid to 70% acetonitrile and 0.2% formic acid. A blank was analyzed between samples to limit carry-over. Data-dependent analysis was utilized on the LTQ to perform MS/MS on all ions above an ion count of 1000. Dynamic exclusion was set to exclude ions from MS/MS selection for 3 min after being selected 2 times in a 30 s window.

### 4.10. Antigen Presentation Assays

CWR22.Vec, CWR22.GILT, CWR22.DR4, CWR22.DR4.GILT, PC-3.vec, and PC-3.DR4 cells (2 × 10^5^ cells/well) were plated for 24 h at 37 °C in culture media in a 96-well flat-bottomed plate. Following incubation with peptides or control buffer, cells were washed in DPBS and fixed with 1% paraformaldehyde. Cells were then washed and cocultured with respective peptide-specific CD4^+^ T cells. In the same assays, cells were incubated with T cells in the presence or absence of anti-class II antibodies L243 as described [42,43]. CWR22Rv1.vec cells were also incubated in the presence of the immunodominant PSMA_459_ peptide in media or HBSS, followed by coculturing with peptide-specific CD4+ T cells. Cell supernatants were analyzed by ELISA for either IL-2 or IFN-γ production according to the manufacturer’s instructions (R&D Systems, Minnieapolis, MN, USA). All assays were repeated at least three times.

### 4.11. Enzyme-Linked Immunosorbent Assay

To analyze cytokines, interleukin-2 and IFN-γ, cell supernatants from triplicate wells from 96-well plates were evaluated by ELISA (R&D Systems, Minneapolis, MN, USA) according to the manufacturer’s instructions [30]. Anti-IFN-γ was purchased from R&D Systems, and anti-IL-2 was purchased from Sigma Aldrich (St. Louis, MO, USA). All assays were repeated at least three times.

### 4.12. Statistics

Data from each experimental group were subjected to statistical analysis. The data were obtained from three independent experiments. The immunoreactive bands of Western blotting were quantified by densitometric analysis using the ImageJ software (U.S. National Institutes of Health, Bethesda, MD, USA). Differences between experimental groups were analyzed for statistical significance using Student’s *t*-test and one-way ANOVA. Data analyses were also performed using the GraphPad Software, and the differences were calculated using one-way ANOVA or Student’s *t* test. Values of *p* ≤ 0.05 were considered significant.

## 5. Conclusions

Prostate cancer is one of the most prevalent cancers among males with an increasing incidence worldwide. Although treatments such as prostatectomies and radiotherapy for localized prostate cancer have yielded good results, similar outcomes have not been achieved for metastatic cancer. Thus, new forms of therapy are urgently needed for aggressive and metastatic prostate cancer. Here, we showed that the insertion of GILT into prostate cancer cells enhanced PSMA processing and presentation via the HLA class II pathway with an increased CD4^+^ T cell recognition of prostate cancer cells. We also showed that GILT insertion did not alter the expression of PSMA, an important target in prostate cancer vaccine strategies. Mechanistic studies showed that the immunodominant PSMA_459_ epitope becomes cysteinylated under normal physiologic conditions, and this cysteinylated form of PSMA_459_ inhibits the CD4^+^ T cell recognition of prostate cancer cells. Taken together, these findings suggest GILT has the potential to increase the HLA class II Ag presentation and activation of T cells. Furthermore, through the processing of immunodominant peptide ligands, more effective T cell activation can be accomplished that may also recognize GILT-negative cells. These functional peptide ligands could then improve the effectiveness of protein/peptide-based prostate cancer vaccine strategies.

## Data Availability

Data will be available upon written request after publication.

## Abbreviations

HLA	Human leukocyte antigen
PSMA	Prostate-specific membrane antigen
GILT	Gamma–interferon inducible lysosomal thiol reductase
IMDM	Iscove’s Modified Dulbecco’s Medium
IFN-γ	Interferon-gamma
PBMC	Peripheral blood mononuclear cells
DTT	Dithiothreitol
ELISA	Enzyme-linked immunosorbent assay
LC	Liquid chromatography
MS/MS	Mass spectrometry
AU	Arbitrary units
ANOVA	Analysis of variance
STDEV	Standard deviation
